# A Crowdsourcing Approach to Develop Machine Learning Models to Quantify Radiographic Joint Damage in Rheumatoid Arthritis

**DOI:** 10.1001/jamanetworkopen.2022.27423

**Published:** 2022-08-29

**Authors:** Dongmei Sun, Thanh M. Nguyen, Robert J. Allaway, Jelai Wang, Verena Chung, Thomas V. Yu, Michael Mason, Isaac Dimitrovsky, Lars Ericson, Hongyang Li, Yuanfang Guan, Ariel Israel, Alex Olar, Balint Armin Pataki, Gustavo Stolovitzky, Justin Guinney, Percio S. Gulko, Mason B. Frazier, Jake Y. Chen, James C. Costello, S. Louis Bridges

**Affiliations:** 1 University of Alabama at Birmingham, Birmingham; 2 Division of Rheumatology, Department of Medicine, Hospital for Special Surgery, New York, New York; 3 Sage Bionetworks, Seattle, Washington; 4 WRQ Research, New York, New York; 5 Catskills Research, Davidson, North Carolina; 6 Department of Computational Medicine and Bioinformatics, University of Michigan, Ann Arbor; 7 Leumit Health Services, Tel-Aviv, Israel; 8 Medical Solutions for Digital Medicine, Jerusalem, Israel; 9 Department of Complex Systems in Physics, Eötvös Loránd University, Budapest, Hungary; 10 T. J. Watson Research Center, IBM, Yorktown Heights, New York; 11 Now with Sema4, Stamford, Connecticut; 12 Division of Rheumatology, Department of Medicine, Icahn School of Medicine at Mount Sinai, New York, New York; 13 Department of Pharmacology, University of Colorado Anschutz Medical Campus, Aurora; 14 Division of Rheumatology, Weill Cornell Medical College, New York, New York

## Abstract

**Importance:**

An automated, accurate method is needed for unbiased assessment quantifying accrual of joint space narrowing and erosions on radiographic images of the hands and wrists, and feet for clinical trials, monitoring of joint damage over time, assisting rheumatologists with treatment decisions. Such a method has the potential to be directly integrated into electronic health records.

**Objectives:**

To design and implement an international crowdsourcing competition to catalyze the development of machine learning methods to quantify radiographic damage in rheumatoid arthritis (RA).

**Design, Setting, and Participants:**

This diagnostic/prognostic study describes the Rheumatoid Arthritis 2–Dialogue for Reverse Engineering Assessment and Methods (RA2-DREAM Challenge), which used existing radiographic images and expert-curated Sharp-van der Heijde (SvH) scores from 2 clinical studies (674 radiographic sets from 562 patients) for training (367 sets), leaderboard (119 sets), and final evaluation (188 sets). Challenge participants were tasked with developing methods to automatically quantify overall damage (subchallenge 1), joint space narrowing (subchallenge 2), and erosions (subchallenge 3). The challenge was finished on June 30, 2020.

**Main Outcomes and Measures:**

Scores derived from submitted algorithms were compared with the expert-curated SvH scores, and a baseline model was created for benchmark comparison. Performances were ranked using weighted root mean square error (RMSE). The performance and reproductivity of each algorithm was assessed using Bayes factor from bootstrapped data, and further evaluated with a postchallenge independent validation data set.

**Results:**

The RA2-DREAM Challenge received a total of 173 submissions from 26 participants or teams in 7 countries for the leaderboard round, and 13 submissions were included in the final evaluation. The weighted RMSEs metric showed that the winning algorithms produced scores that were very close to the expert-curated SvH scores. Top teams included Team Shirin for subchallenge 1 (weighted RMSE, 0.44), HYL-YFG (Hongyang Li and Yuanfang Guan) subchallenge 2 (weighted RMSE, 0.38), and Gold Therapy for subchallenge 3 (weighted RMSE, 0.43). Bootstrapping/Bayes factor approach and the postchallenge independent validation confirmed the reproducibility and the estimation concordance indices between final evaluation and postchallenge independent validation data set were 0.71 for subchallenge 1, 0.78 for subchallenge 2, and 0.82 for subchallenge 3.

**Conclusions and Relevance:**

The RA2-DREAM Challenge resulted in the development of algorithms that provide feasible, quick, and accurate methods to quantify joint damage in RA. Ultimately, these methods could help research studies on RA joint damage and may be integrated into electronic health records to help clinicians serve patients better by providing timely, reliable, and quantitative information for making treatment decisions to prevent further damage.

## Key Points

**Question:**

Can a worldwide collaborative effort develop machine learning algorithms to quantify joint space narrowing and erosions automatically to improve the current visual inspection approach to radiography in rheumatoid arthritis (RA)?

**Findings:**

This prognostic study assesses an international, crowdsourcing competition using scored radiographs of hands/wrists and feet from 3 clinical studies of patients with RA to develop machine learning algorithms to quantify damage in RA. The accuracy and reproducibility of the submitted algorithms were confirmed with a postchallenge independent validation data set.

**Meaning:**

These findings suggest that after refining and validating with larger cohorts, these algorithms alone or in combination could be incorporated into electronic health records, contributing to more informed and precise management of RA.

## Teaser

This diagnostic/prognostic study presents assesses machine learning algorithms submitted to an international crowdsourcing contest to develop methos to automatically quantify radiographic evidence of damage in patients with rheumatoid arthritis (RA).

## Introduction

Rheumatoid arthritis (RA) is a chronic inflammatory disease with a global prevalence estimated to be 0.24% in 2010. The prevalence is approximately 2-fold higher in women than men.^1^ The hallmark of RA is inflammation in the synovial lining of joints, leading to joint space narrowing (JSN) and erosions in subchondral bone, joint deformity, and, often, disability.^2^ Not all patients with RA develop joint damage, which reflects variability in causes and pathogenesis of the disease. In addition, some patients with minimal symptoms can slowly develop progressive damage over time that is only identified via radiographic examination and may not be readily recognized by clinicians owing to its insidious nature.

Determining whether a patient is accruing joint damage and assessing the rate of progression are major challenges in RA. Real-time quantitative, objective assessment of damage via radiography and comparison with previous images may thus guide escalation of therapy in patients with ongoing joint damage. Although advanced imaging techniques (eg, small coil magnetic resonance imaging, ultrasonography) can help visualize joint damage, these techniques are either expensive, unavailable to many patients, or operator dependent (ie, subjective and dependent on the skill of the person acquiring the images). After the diagnosis of RA is confirmed, quantifying RA-related bone and joint damage is conducted by assessing radiographs of joints in the hands, wrists, and feet, the joints typically affected in RA, and scoring of the degree of JSN and erosion in each of these joint areas. The Sharp-van der Heijde (SvH) method^3^ is currently the generally most accepted scoring system for RA. However, this approach is time-consuming, labor-intensive, and requires specialized training. Researchers studying clinical outcomes of RA would benefit from an automated method to assess the degree of joint damage quickly, accurately, and reproducibly. Such a method would allow thousands of images currently in electronic health records (EHRs) worldwide to be rapidly scored and used for clinical research studies. After clinical validation, such methods could be integrated into EHRs for reliable, efficient monitoring of joint damage progression, which could lead to better therapeutic decisions (eg, changing disease modifying antirheumatic drugs that are not controlling damage detected via radiography).

**Figure 1.**
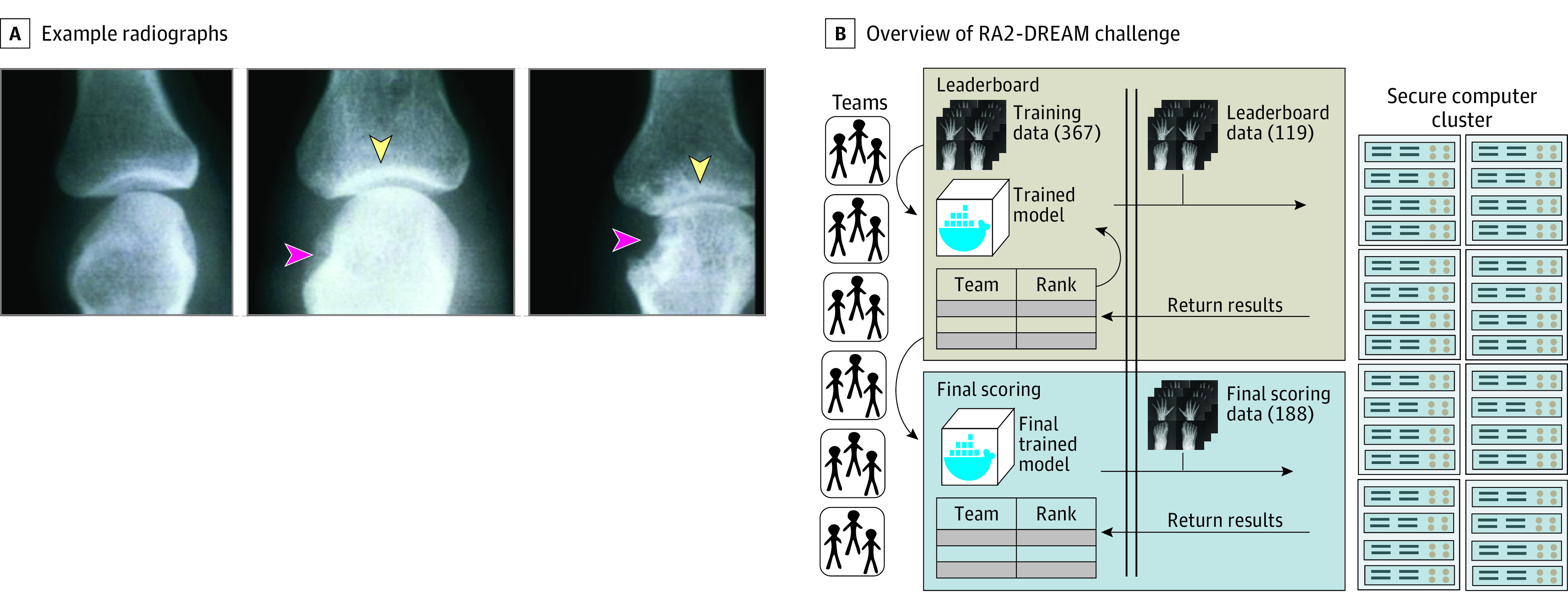
An Overview of RA2-DREAM Challenge A. Representative radiographs showing joints without disease, joints with mild and severe damages due to RA (copyright 2022 ACR). Red arrows indicate areas of erosion; yellow arrows, areas with joint space narrowing. B. A total of 367 sets of radiographic images and expert-curated SvH scores were provided to participants to train algorithms. Leaderboard data set (119 images only) and final evaluation data set (188 images only) were used for performance evaluation in the leaderboard and final evaluation rounds.

Algorithms based on deep learning (DL) have attained human-level or even better performance in image classification, as shown in ImageNet competitions.^4,5^ For example, DL methods have been studied to aid the diagnosis of pulmonary tuberculosis or pneumonia from chest radiographs.^6^ RA-related JSN and erosions of cortical bone represent a unique challenge owing to the numerous joints involved (eg, proximal interphalangeal, metacarpophalangeal, and many joints in the wrist) and the complex anatomy (eg, joints in the wrist are composed of multiple bones) (Figure 1A). While a number of studies have made advances in building machine learning models for scoring radiographs,^7,8,9^ no independently benchmarked, accessible, and automated methods to quantify RA-associated joint damage are available, to our knowledge.

The Rheumatoid Arthritis 2–Dialogue for Reverse Engineering Assessment and Methods (RA2-DREAM) Challenge, a worldwide collaborative competition, engaged a diverse community of biologists, computer scientists, and physicians. Teams that participated in the challenge were provided with access to high-resolution radiographs of hands, wrists, and feet from 2 studies funded by the National Institutes of Health in which criterion standard SvH scores were already available: Bridges et al^10^ and Ptacek et al.^11^ We received a total of 173 submissions from 26 participants or teams in 7 countries for the leaderboard and 13 submissions for final evaluation. Teams were ranked in each subchallenge according to the performance of their algorithms. Reproducibility of the submitted methods was evaluated by subsampling the final evaluation data set and rescoring methods (ie, bootstrapping). All algorithms were then validated using a postchallenge independent data set from the Treatment of Early Aggressive Rheumatoid Arthritis (TEAR) trial.^12^ The winning algorithms achieved performances that were significantly better than the baseline model and very close to expert-curated SvH scores. This supports the hypothesis that automated scoring of RA radiographs is a feasible and promising approach that may be used in research studies with the goal of moving into clinical practice.

## Methods

This diagnostic/prognostic study was approved by the University of Alabama at Birmingham (UAB) institutional review board, including use of the Challenge data. The requirement for informed consent was waived according to 45 CFR 46.116. Individual write-ups, Docker containers, and other documentation are available elsewhere.^13^

### RA2-DREAM Challenge Data

The RA2-DREAM Challenge used radiographic images and standard SvH scores^14^ from 2 NIH-funded clinical studies, Bridges et al^10^ and Ptacek et al,^11^ led by investigators at the UAB. A total of 674 sets of images with scores from 562 patients were used with some patients’ data at 2 time points (Figure 1). Patient demographic data, including self-reported race, were collected during enrollment. Race was categorized as Black, White, or other (including American Indian or Alaska Native, Asian, and Native Hawaiian or other Pacific Islander) and was included because it was available in each parent research study. Each radiographic set included 4 images: 1 from each foot and 1 from each hand and wrist, all taken in a posteroanterior direction. The SvH scoring system assesses JSN on 15 areas from the hand and wrist and 6 areas from the foot (range, 0-4 for each joint) and erosions from 16 joint areas of the hand and wrist (range, 0-5 for each joint) and 2 sides of 6 joints of the foot (range, 0-10 for each joint), resulting in a total score of 448. Importantly, some participants had no RA-related joint damage of the hands and wrists or feet.^10,15^ Individual joints with no damage attributable to RA were used as controls (SvH score, 0) for algorithm training.

All sets of images and scores were allocated to training (367 radiographic images and SvH scores), leaderboard (119 images only), and final evaluation (188 images only) data sets. The weight-bin scheme was chosen to balance the number of patients of certain ranges of SvH scores in each database and also to decrease the amount of influence of very high or very low SvH scores would have, given that SvH scores range from 0 to 448. First, we set up 8 bins of SvH scores (or ranges: 0, 1, 2-3, 4-7, 8-20, 21-55, 56-148, and >148). The threshold of each bin (0, 1, 1.1, 1.95, 3, 4, 5, and 6) was defined by the log value of the upper limit of each bin, with the exceptions to the first 2 bins (score 0, indicating no damage, and score 1). We then use the weight (*w_i_*) of 2^bin_threshold^ to generate equal-weighted date sets.

### Challenge Procedures

The RA2-DREAM Challenge contained 3 subchallenges. Participants were tasked to develop automated methods to quickly and accurately quantify overall RA damage (subchallenge 1), JSN (subchallenge 2), and erosion (subchallenge 3) from radiographic images of hands and feet. To reach the goals, the challenge had 3 rounds: training, leaderboard, and final evaluation (Figure 1B). In the training round, teams were provided with a training data set to develop computational methods to quantify RA-related damage in typically affected joints. In the leaderboard round, teams refined their algorithms with the leaderboard data set through sequential submissions and feedback on performance scored against expert-curated SvH scores. Each team was allowed up to 3 submissions per week over 9 weeks, totaling 27 potential submissions (eFigure 1 in Supplement 1). Public leaderboards with weighted-RMSE were updated immediately after submission and evaluation. This helped teams to gain immediate feedback on their algorithms and modify them accordingly. In the final evaluation round, 2 submissions per team were allowed, and the final submission, including a written description, was evaluated to determine the team’s ranking in each subchallenge.

All teams submitted their models via the Synapse collaborative science platform (Sage Bionetworks) (eMethods in Supplement 1). Models submitted to the platform were automatically transferred to the UAB Cheaha supercomputer and executed on the challenge data to produce estimation files (Figure 1B). The challenge was finished on June 30, 2020.

### Evaluation of the Performance of Algorithms

We assessed the performance of each team’s model by comparing teams’ scores of each joint (*y_i_*) to the ground truth SvH scores (*s_i_*) using a patient-weighted RMSE approach. To avoid minus infinity (log zero) of a healthy joint we added 1 to *y_i_* and *s_i_* before log transformation using the equation:



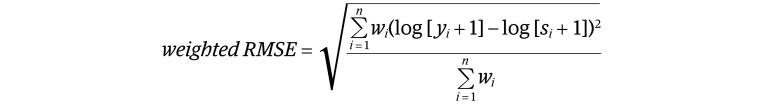



### Baseline Model

A baseline model^16^ was developed by the RA2-DREAM Challenge organizers for comparison to the models submitted. We created the baseline model by training a 10-layer DL model without segmentation of the images to classify the damage of each joint. The subchallenge 2 and subchallenge 3 scores were summed to generate subchallenge 1 scores for the baseline model. The layered architecture followed the example described in *Deep Learning with Python*.^17^

### Postchallenge Independent Validation

To validate the performance of all algorithms, we selected 50 sets of images and scores reflecting various degrees of damage from the TEAR trial.^12^ In the TEAR trial, there were 2 sets of SvH scores for each set of images, generated by 2 independent readers. We ran all submitted models on the 50 sets of images and evaluated their performance against the mean SvH scores of the 2 readers. We also computed the Spearman correlation between the final evaluation and postchallenge independent validation data set to assess the reproducibility of the top-performing algorithms. The concordance index between the final evaluation and postchallenge independent validation was calculated using the concordance_index function in the Python lifelines library.^18^ A false discovery rate–corrected pairwise *t* test was also performed as a secondary comparison of performance for each team relative to the top-performing team in each subchallenge.

### Reproducibility of Methods Evaluated by Bootstrapping

The reproducibility of submitted algorithms and the baseline model was determined using Bayes factor calculated for 1000 bootstrapped iterations^19^ of the final evaluation and postchallenge independent data sets. This analysis identified the methods that are better than, tied with, or worse than a reference method considering the performance on a random sampling of the data. In each subset, we reran all the algorithms to obtain scores for subchallenge 1, subchallenge 2, and subchallenge 3. We then used these scores to calculate Bayes factors for each subchallenge using the computeBayesFactor function of R package challengescoring^20^ in comparison with the top-performing model using R statistical software version 4.2.1 (R Project for Statistical Computing).^21,22,23,24,25,26^

### Ensemble Modeling

As with previous DREAM Challenges, we explored the so-called *wisdom of the crowds* phenomenon by performing ensembling experiments using the models submitted in the final evaluation round.^21,22,23,24,25,26^ For each subchallenge, we aggregated the top performer alone, the top 2, the top 3, and so on, then evaluated these ensemble estimations by calculating the mean estimation for each joint across multiple algorithms using the previously described Bayes factor robustness analysis.

### Statistical Analysis

For comparison of demographic characteristics, we reported mean (SD) or median (IQR) for continuous variables. Spearman correlation, 2-sided pairwise *t* test, Bayes factor calculation, and Kruskal-Wallis test were performed using Python or R packages. *P* values were 2-sided, and *P* < .05 was considered as significant. Data were analyzed from June 24, 2020, to September 7, 2021.

## Results

### Selection of Top-Performing Teams for Subchallenges

All 3 subchallenges were evaluated separately based on the assumption that algorithms to score JSN may be different from those used to score erosions (Figure 1A) and that there may be an algorithm that could give an overall quantification of damage for both parameters. Patient sociodemographic and clinical characteristics used for the challenge and postchallenge independent validation are shown in Table 1. The mean (SD) age was 54.9 (13.2) years in the training data set, 51.4 (13.1) years in the leaderboard data set, and 53.5 (13.5) years in the final evaluation data set. Data sets included mostly women, with 307 (83.7%) women in the training data set, 108 (90.8%) women in the leaderboard data set, and 158 (84.0%) women in the final evaluation data set. These demographic characteristics are very consistent with typical populations of RA, which predominantly affects women.

**Table 1.** Demographic Characteristics of Patients in Training, Leaderboard, and Final Evaluation Data Sets

Characteristic	Data set, No. (%)		
	Training (n = 367)	Leaderboard (n = 119)	Final evaluation (n = 188)
Score, median (IQR)			
Overall total	13.0 (5.0-38.5)	5.0 (2.0-15.5)	10.0 (5.0-20.0)
Joint erosion	5.0 (2.0-17.0)	2.0 (1.0-5.0)	5.0 (2.0-9.0)
Joint narrowing	7.0 (2.0-22.5)	2.0 (0.0-11.0)	4.0 (0.8-11.3)
Sex			
Men	60 (16.3)	11 (9.2)	30 (16.0)
Women	307 (83.7)	108 (90.8)	158 (84.0)
Race			
Black	242 (65.9)	88 (73.9)	156 (83.0)
White	106 (28.9)	26 (21.8)	21 (11.2)
Other^a^	19 (5.2)	5 (4.2)	11 (5.9)
Age, mean (SD), y			
At radiographic examination	54.9 (13.2)	51.4 (13.1)	53.5 (13.5)
At RA diagnosis	46.3 (13.3)	45.9 (13.9)	48.4 (13.1)
Disease duration, mean (SD), mo	103.2 (113.1)	65.4 (74.3)	60.5 (93.9)
Rheumatoid factor^b^			
Positive	273 (74.4)	89 (74.8)	126 (67.0)
Negative	90 (24.5)	29 (24.4)	60 (31.9)
NA	4 (1.1)	1 (0.8)	2 (1.1)
Anti-CCP antibody^b^			
Positive	250 (68.1)	76 (63.9)	112 (59.6)
Negative	94 (25.6)	39 (32.8)	68 (36.2)
NA	23 (6.3)	4 (3.4)	8 (4.2)

Abbreviations: CCP, cyclic citrullinated peptide; NA, not available; RA, rheumatoid arthritis.

^a^ Includes American Indian or Alaska Native, Asian, and Native Hawaiian or other Pacific Islander individuals.

^b^ Rheumatoid factor and anti-CCP antibodies are 2 autoantibodies characteristic of RA.

**Figure 2.**
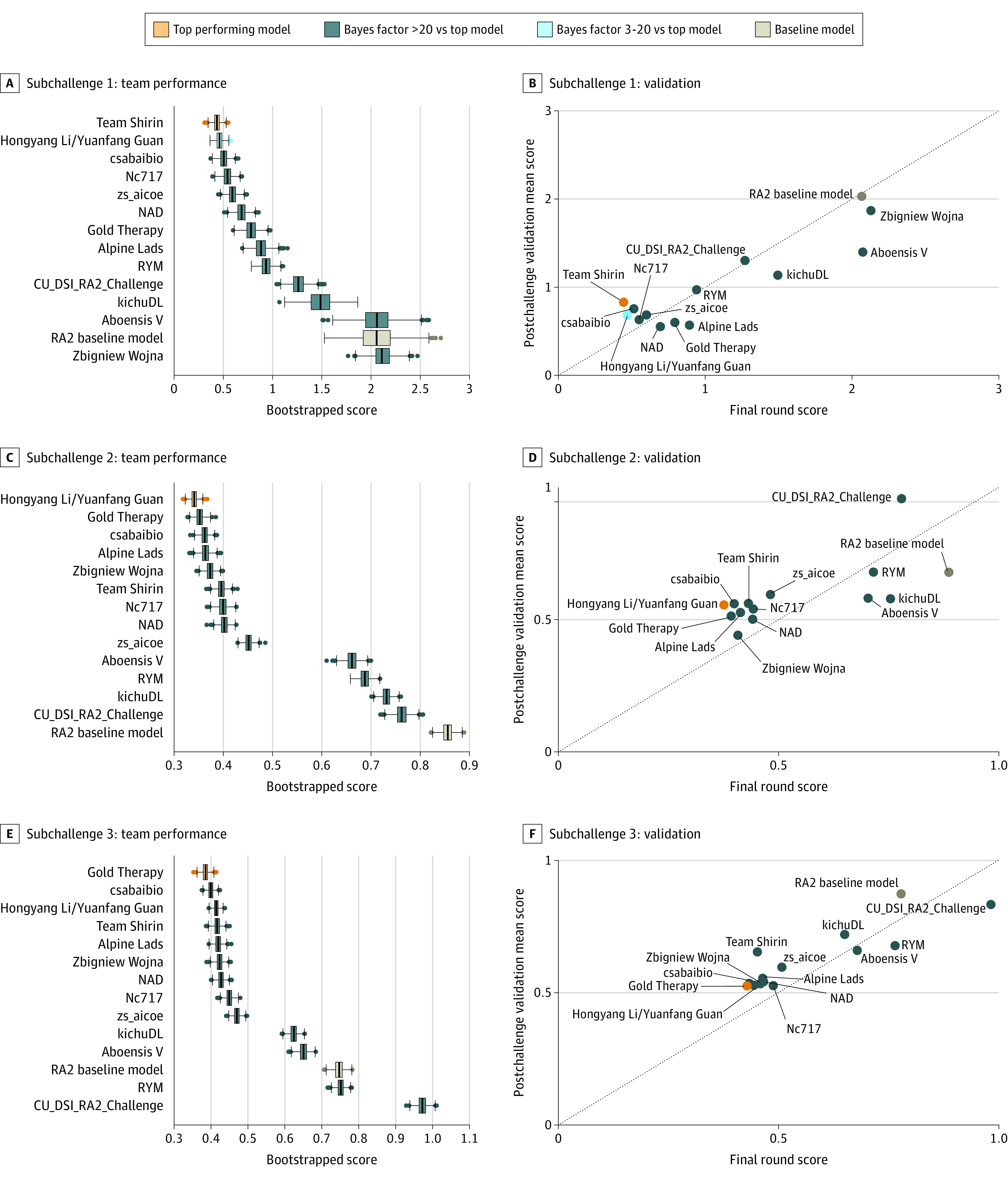
Evaluation and Validation of Challenge Results All estimations were bootstrapped to generate a distribution of scores to calculate Bayes factors between the top performing model and all other models. Any estimation with a Bayes factor of 3 or less was considered “tied” with the top model; no estimations were tied for top place in any of the 3 subchallenges. The baseline model provided by the organizers was used for reference. The models were run on postchallenge independent validation data set of 50 images and scored against mean of the 2 expert-curated measurements from the validation data set, using the overall damage (subchallenge 1), joint space narrowing (subchallenge 2), and erosion (subchallenge 3) metrics. Left, Bold lines indicate medians; boxes, IQRs; whiskers, ranges; and dots, individual data points. Right, The dot plots show the weighted RMSE performance in the final evaluation round (x-axis) for each model compared with the mean of weighted RMSE (2 readers) from the validation data set (y-axis). Algorithms below the dashed line performed better in the final evaluation round, while those above the dashed line performed better on the validation data set.

The accuracy of each algorithm was assessed using the weighted RMSE metric, comparing the estimated scores with the expert curated SvH scores. The reproducibility of each submitted algorithm was evaluated using Bayes factor. Briefly, the final evaluation data set was subsampled, algorithms were scored on the subsampled data set, and Bayes factor was calculated after 1000 bootstrapped iterations. A Bayes factor greater than 3 was used to determine if a team’s algorithms outperformed another team’s algorithm; no ties were observed (Figure 2). Top performing teams across all subchallenges had better performance than the baseline model. Teams Shirin and HYL-YFG (Hongyang Li and Yuanfang Guan) were the top 2 ranked teams for subchallenge 1; teams HYL-YFG, Gold Therapy, and csabaibio were the top 3 for subchallenge 2; and teams Gold Therapy, csabaibio, and HYL-YFG were the top 3 for subchallenge 3 (Figure 2; eFigure 2 and eFigure 3 in Supplement 1). They were awarded a total of $50 000 in prize money (eMethods in Supplement 1). The RMSE measures how closely the estimated values from the submitted algorithm were to the known SvH scores. The RMSE of the baseline model was 4.65 for subchallenge 1, 0.71 for subchallenge 2, and 0.65 for subchallenge 3, while the lowest RMSEs were 0.44 for subchallenge 1, 0.38 for subchallenge 2, and 0.43 for subchallenge 3. Detailed descriptions of algorithm development from the teams are provided in the eMethods in Supplement 1.

### Rigor and Reproducibility of Top-Performing Algorithms

After completion of the challenge, we validated the consistency of algorithms using the postchallenge independent validation data set from the TEAR study,^12^ which contained lower-quality radiographs than those from the studies by Bridges et al^10^ and Ptacek et al,^11^ suggesting that the postchallenge independent validation task was likely more difficult. We calculated the weighted-RMSE and compared algorithms’ performance with those in the final evaluation round (Figure 2). We also computed a concordance index ranging from 1 (same ordering) to 0.5 (random ordering) to 0 (inverse ordering) between the scores in the final evaluation round and those in postchallenge independent validation. The concordance indices were 0.71 for subchallenge 1, 0.78 for subchallenge 2, and 0.82 for subchallenge 3, suggesting that the methods were reasonably generalizable and these algorithms can be readily applied to new independent radiographic images.

There were some inconsistencies in top-performing algorithms between the final evaluation and postchallenge independent validation. For example, Team NAD in subchallenge 1 and Zbigniew Wojna in subchallenge 2 showed better performance than others in the postchallenge independent validation, suggesting these models may perform better than others in images with different resolution or quality or in data sets with differences in which joint areas had more damage, such as wrists vs proximal interphalangeal joints. These results suggest that combinations of algorithms may be more robust than individual models alone, which led us to develop and evaluate an ensemble approach.

### Ensemble Models vs Individual Models

**Figure 3.**
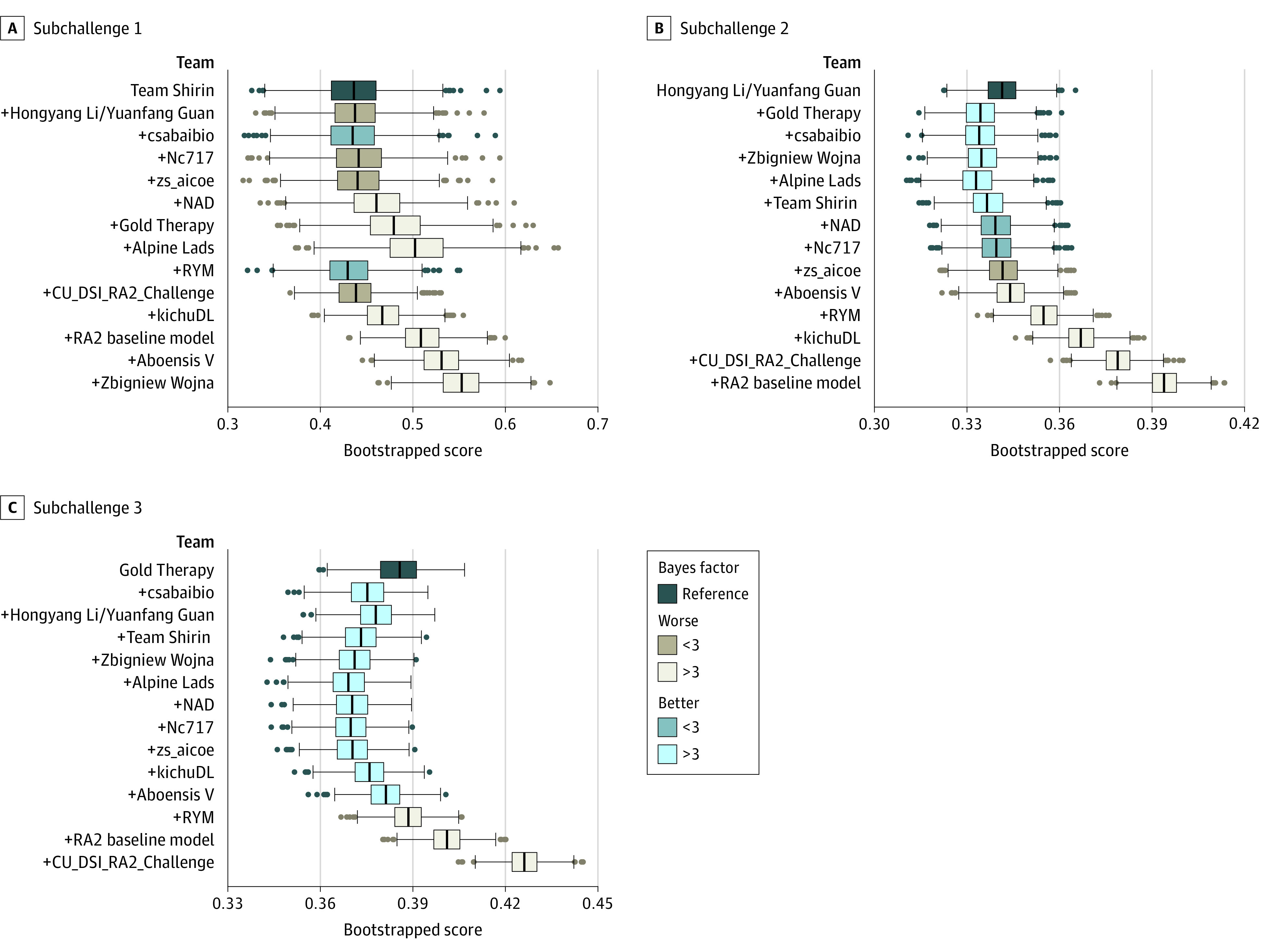
Ensembled Models Improve Performance (Weighted RMSE) A series of ensemble models were created by combining the top 2 models, the top 3, and so on until all models were combined (from x-axis left to right, model appears according to its ranking in each subchallenge). For each ensemble model, the means of the estimations were calculated and scored with the overall damage (subchallenge 1), joint space narrowing (subchallenge 2), or erosion (subchallenge 3) metrics. A bootstrap Bayes factor analysis was used to determine differences in performance between the top-performing (individual) model and the ensemble models.

We calculated the means of estimations from the top 2 performing algorithms then added the following ranked teams (Figure 3). We performed the same bootstrap analysis as was performed for individual teams to assess the reproducibility of the ensemble models compared with the top-performing team measured by Bayes factor.

For subchallenge 1 (overall damage), the ensembled models were not significantly better than the top-performing method (Bayes factor >3) (Figure 3A). Interestingly, including estimations from team RYM substantially improved performance in subchallenge 1, although this algorithm was not one of the top-ranked models in subchallenge 1. We further explored this by evaluating the pairwise Spearman correlation for all individual final evaluation round estimations in subchallenge 1, subchallenge 2, and subchallenge 3 (eFigure 3 in Supplement 1). Notably, in subchallenge 1, team RYM had a substantially lower Spearman correlation (ρ = 0.65-0.73) with the better-performing algorithms than the pairwise Spearman correlations within the better-performing algorithms (ρ = 0.75-0.95) (eFigure 3 in Supplement 1). This suggests that the algorithm from team RYM may improve the ensemble by providing information that is orthogonal to the other top estimations, a phenomenon that has been previously observed.^27^ After evaluating the distribution of the estimations, we found that the ensemble model that included the top 9 teams systematically performed better than the top-performing team (eFigure 2 in Supplement 1). In subchallenge 2 and subchallenge 3, estimations were less concordant between the top teams than they were in subchallenge 1 (eFigure 3 in Supplement 1). The mean of the estimations from several of the top-ranked models (up to 5 models for subchallenge 2 and up to 10 models for subchallenge 3) increased the estimation power of the algorithms substantially (Figure 3B and C; eFigure 2 and eFigure 4 in Supplement 1). The estimation tasks for JSN and erosions improved (subchallenge 2 and subchallenge 3) more with ensembling than the estimation task of overall damage (subchallenge 1). We also scored the ensemble estimations shown in Figure 3 using the Spearman correlation as an outcome metric, which showed similar results to the weighted RMSE (eFigure 4 in Supplement 1). Taken together, we found that ensembling top models yielded similar improvements in performance in all 3 subchallenges.

### Impact of Individual Technical Approaches on Team Performance

To gain insights into the submitted models and aid refinements of these and other imaging algorithms, we systematically summarized the top-performing teams’ methods by conducting a postchallenge survey and evaluating the methodological approaches (Table 2).^28,29,30,31,32,33,34^ Of 13 teams included in final evaluation, 10 applied image segmentation before attempting to quantify damage. In general, teams that applied image segmentation achieved better performance for all 3 subchallenges (eMethods and eFigure 5 in Supplement 1), although the difference between using and not using segmentation was not statistically significant.

**Table 2.** Summary of Machine Learning Methods Used by Teams Who Submitted Models in the Final Evaluation Round

Team name	Segmentation model	Image augmentation	Algorithm class	Prebuild models	Ensemble models, No.
Team Shirin	NA^a^	No	Other	DenseNet^28^	NA^b^
HYL-YFG	U-Net^29^	No	ConvNet	NA^c^	10
Gold Therapy	ResNet^30^	No	ConvNet	NA^c^	NA^b^
CSAbaibio	RCNN,^31^ ResNet^30^	No	ConvNet	NA^c^	12
Nc717	RetinaNet^32^	No	ConvNet	EfficientNet^33^	5
Aboensis V	YOLO^34^	No	ConvNet	YOLO^34^	NA^b^
NAD	RCNN^31^	No	ConvNet	EfficientNet^33^	15
kichuDL	NA^a^	No	Autoencoder	NA^c^	20
Alpine Lads	NA^a^	Yes	ConvNet	NA^c^	2
Zbigniew Wojna	U-Net^29^	No	ConvNet	NA^c^	8
RYM	YOLO^34^	Yes	PenReg	NA^c^	NA^b^
CU_DSI^d^	RCNN^31^	No	ConvNet	EfficientNet	NA^b^
ZS_ai^d^	YOLO^34^	Yes	ConvNet	ResNet,^30^ DenseNet^28^	3

Abbreviations: HYL-YFG, Hongyang Li and Yuanfang Guan; NA, not available.

^a^ The team did not apply segmentation.

^b^ The team did not use an ensemble model.

^c^ The team did not apply any prebuilt model.

^d^ Response not received but information was extracted from write-ups.

Of 13 teams in the final evaluation, 10 applied DL-based approaches. The mean scores among algorithms that used DL were not statistically significantly different (eFigure 5 in Supplement 1). Nor did we did find that teams that used ensemble approaches had more favorable scores (eFigure 5 in Supplement 1).

After the challenge, we performed visual examination of images in which there were significantly discordant scores between expert SvH and algorithm-generated scores (eMethods and eFigure 6 in Supplement 1). From individual joints in the final evaluation data set, we noted 201 outliers of 7896 JSN scores (2.5%) and 462 outliers of 8272 erosion scores (5.6%). One of the coauthors, a board-certified musculoskeletal radiologist (M.B.F.) rescored the outliers by visual inspection according to SvH methods and made corrections to 97 SvH JSN scores (1.2%) and 192 erosion scores (2.3%). The low number of revised scores suggests a high degree of accuracy in the SvH scores generated by the trained scorers. Two expert readers generated the original SvH scores in the TEAR trial.^12^ The coefficients of variation between the 2 expert readers were 0.74 for overall scores (*P* = .15), 0.62 for JSN scores (*P* = .18), and 0.73 for erosion scores (*P* = .03). Together these results suggested that scoring erosion is more challenging than scoring JSN.

## Discussion

In this prognostic study, we leveraged an international community of biomedical, computer science, and engineering experts to develop methods to quantify RA disease severity from radiographs of the hands, wrists, and feet through machine learning techniques. Although there is complexity and variability inherent in the images from patients, the top-performing algorithms achieved relatively high accuracy and were reproducible. We made several observations that were in line with expectations. First, scoring of the metacarpophalangeal and proximal interphalangeal joints in the hands and forefoot were more accurate than those in the wrist. This can likely be explained by the anatomic complexity of the articulations of the 8 carpal bones; posteroanterior images lead to difficulty in visualizing all components of the joints. Second, we found that scores for JSN were more concordant with SvH scores than those for erosions. JSN may be more straightforward because it involves measuring distances, while identification of erosions depends on bone morphological characteristics and their disruption.

As shown in the postchallenge evaluation of discordant scores, assessing joint erosions is more difficult than assessing JSN both in human readers and in algorithms. Visualizing the bony cortex precisely (which is part of identifying erosions) is difficult owing to the complexity of the anatomical relationships. Future effort to develop automated optimization of images through digital manipulation of brightness, contrast, and rotation may overcome this difficulty.

Most of the RA2-DREAM Challenge participants used DL-based methods, reflecting a general trend in image analysis research and the freely accessible extendable architecture of pretrained models (eg, DenseNet,^28^ ResNet,^30^ and U-Net^29^). Thus, final algorithm performance may depend more on other technical decisions rather than use of a DL framework. Importantly, building ensemble models substantially improved the estimation performance and is likely a strategy to be included in future development.

These approaches may also be applied to the vast number of images in data warehouses and EHRs to greatly improve the statistical power of research studies designed to identify factors associated with RA damage and its progression. Significantly expanding our capability to study joint damage at a lower cost and with greater reproducibility creates new possibilities for understanding the pathogenesis of RA and the development of new strategies to prevent joint damage.

Eventually, refined models that have been deployed, optimized, and validated within the research workflow may be directly integrated into the EHR.^35^ A path for the acquired images to be sent directly from a Digital Imaging and Communications in Medicine router to the artificial intelligence (AI) model would be created, received images would be processed using the verified model, and results from the model would then become part of a patient’s EHR via the picture archiving and communication system. The AI results would be denoted properly to indicate that they were generated by the AI system and not by human interpretation. This system would also permit a set of images to be flagged for review by a radiologist. Thus, the successful implementation of automated scoring by algorithms could make the quantified assessment available rapidly to help clinicians make therapeutic decisions. It may also prove valuable in medical communities without musculoskeletal radiologists. By providing feedback on progression of damage quickly, clinicians may be alerted to change treatment regimens to avoid additional joint damage.

Lessons learned in RA may contribute to quantification of radiographic damage in other types of arthritis in which patterns of damage are different. This might include pencil-in-cup deformities in psoriatic arthritis or cortical erosions with overhanging edges in gout.

### Limitations

This study has some limitations. Our results provide optimism for the future automated scoring, but overall performance is limited by the number of images used in the training. For algorithms to become truly robust, thousands of digitally acquired, annotated images from large-scale observational studies, clinical trials, and EHRs should be used for training, and models should be continuously benchmarked on new data.

## Conclusions

The findings of this prognostic study of the RA2-DREAM Challenge suggest that international, award-incentivized, and crowdsourced collaboration could create robust and reproducible algorithms to interpret radiographic images of bones and joints. Such algorithms have great potential for improving outcomes in patients with RA and other chronic forms of arthritis.

## Notes

Supplement 1.
eMethods.

eReferences.
**eFigure 1.** RA2-DREAM Challenge Timeline **eFigure 2.** Distribution of Individual and Ensembled Predictions **eFigure 3.** Spearman Correlation Between Top Final Round Predictions **eFigure 4.** Performance of Ensembled Models **eFigure 5.** Summary of Approaches and Methods Used By Participated Teams in 3 Subchallenges **eFigure 6.** Identification and Correction of Criterion Standard Outliers

Supplement 2.
The RA2-DREAM Challenge Community Members
