# Supplementary material for: A Crowdsourcing Approach to Develop Machine Learning Models to Quantify Radiographic Joint Damage in Rheumatoid Arthritis
Source: JAMA Netw Open. 2022 Aug 29;5(8):e2227423. doi: 10.1001/jamanetworkopen.2022.27423 (PMC9425151; doi:10.1001/jamanetworkopen.2022.27423)
Supplement: Supplement 1. — eMethods. eReferences. eFigure 1. RA2-DREAM Challenge Timeline eFigure 2. Distribution of Individual and Ensembled Predictions eFigure 3. Spearman Correlation Between Top Final Round Predictions eFigure 4. Performance of Ensembled Models eFigure 5. Summary of Approaches and Methods Used By Participated Teams in 3 Subchallenges eFigure 6. Identification and Correction of Criterion Standard Outliers [file jamanetwopen-e2227423-s001.pdf]

Supplemental Online Content

Sun D, Nguyen TM, Allaway RJ, et al; RA2-DREAM Challenge Community. A crowdsourcing approach to develop machine learning models to quantify radiographic joint damage in rheumatoid arthritis. *JAMA Netw Open*. 2022;5(8):e2227423. doi:10.1001/jamanetworkopen.2022.27423

eMethods.
eReferences.
eFigure 1. RA2-DREAM Challenge Timeline
eFigure 2. Distribution of Individual and Ensembled Predictions
eFigure 3. Spearman Correlation Between Top Final Round Predictions
eFigure 4. Performance of Ensembled Models
eFigure 5. Summary of Approaches and Methods Used By Participated Teams in 3 Subchallenges
eFigure 6. Identification and Correction of Criterion Standard Outliers

This supplemental material has been provided by the authors to give readers additional information about their work.

© 2022 Sun D et al. *JAMA Network Open.*

eMethods.

Submission of Algorithms

All algorithms were submitted in the form of a Docker container. Docker is a software platform that allows code developers to save all of the necessary operating system information, code dependencies, and versioning information in one “container”. This container can then be run on the Docker platform irrespective of operating system; Containers were required to follow a prescribed format to enable automated execution in UAB Cheaha supercomputer. Additionally, the models were required to run without a network connection and had to define an ENTRYPOINT to run the algorithm. Participants trained their models locally and submitted a trained model or an untrained model to be trained at run-time.

Awarding of Prize Money

Total prize money awarded was $50,000 ($10,000 for subchallenge 1; $20,000 for subchallenge 2; and $20,000 for subchallenge 3). After evaluation, we selected the top two performing algorithms (Teams Shirin and HYL-YFG) for subchallenge 1 and awarded prizes of $6,000 (first place) and $4,000 (second place). We selected the top three performing algorithms for subchallenge 2 (HYL-YFG, Gold Therapy, and csabaibio) and subchallenge 3 (Gold therapy, csabaibio, and HYL-YFG). In subchallenges 2 and 3, $10,000 was awarded to the first place team, and $5,000 for second and third place teams.

Spearman correlation of final round predictions

Final round prediction vectors for SC1, SC2 and SC3 were compared by calculating the pairwise Spearman correlation for all predictions for all teams within each subchallenge. The pairwise Spearman correlation was plotted using the "pheatmap" function from the pheatmap R package.

Evaluating the impact of final-round teams' technical decisions on the performance

To better understand the submitted methods to aid future algorithm development and software development, we sent a post-challenge survey to all participating teams and analyzed the results by grouping teams’ scores based on their responses to each question. We then applied the Kruskal-Wallis (KW) test among the groups to evaluate whether the different methodological choices were significantly associated with performance (Table 2 and Figure S5). The survey questions were:

© 2022 Sun D et al. *JAMA Network Open.*

- Which pre-built model the teams used in the segmentation? The options include NA (not applying segmentation), self-built, or use other pre-built software libraries (provide name).
- Whether the team enriched the 'rare class', i.e., severe patients, in the image augmentation step? Image augmentation is the common processing step in image recognition, when synthetic images are created to increase the number of training data points.
- Which class of algorithms the teams applied in predicting the joint damage score? The options include Linear regression, Penalized regression (LASSO, Ridge, etc), deep learning, decision tree - random forest, and others.
- Which pre-train models and software library the teams used to predict the joint damage score?
- How many sub-models the teams built when using the ensemble approach? Briefly, the ensemble approach builds many prediction models for the same task; the final prediction is the aggregation of all sub-models' results.

Computational identification of gold standard outliers

SvH scoring of radiographs by visual inspection is a labor-intensive process and can be error-prone. One strategy for identifying these errors is to compare a collection of algorithm-predicted SvH measurements to visual inspection measurements from the same radiograph. For a given joint score, if the predicted scores from many good models (from challenge participants) are relatively similar and the manual SvH score is an outlier to the predicted scores, then this joint score is more likely an error. We used this concept to identify potential outliers or measurement errors in the final evaluation dataset (Figure S6). We used a binning strategy (Figure S6a-c) to enable the calculation of a false discovery rate (FDR)-corrected empirical p-value (Figure S6d-f) for each gold standard (SvH) measurement in the final evaluation dataset. We binned each individual measurement (total overall, JSN and joint erosion scores) for each joint based on the mean.

After the challenge was completed and for each subchallenge, we took the mean of the top 8 predictions for a given overall score or joint score and assigned each joint to a bin based on this predicted score using the “cutr” R package smart_cut function to generate equal sized bins. We calculated an empirical p-value for each gold standard SvH measurement relative to the other values in the same bin. We then FDR-corrected (false discovery rate) the p-values.

© 2022 Sun D et al. *JAMA Network Open.*

Potential outlier gold standard measurements were defined as those that had an FDR-corrected p-value < 0.1. A board-certified musculoskeletal radiologist reviewed these flagged joints to determine whether the original measurements were inaccurate. If the review indicated that the measurement was incorrect, we re-scored these joints for JSN and erosion (Figure S6d-f, pink bars). We did not identify any putative errors in the gold-standard scores used for SC1 (overall damage) (Figure S6d). For SC2 and SC3, we determined that 201/7896 (2.5%) and 462/8272 (5.6%) scores, respectively, were possible errors in the gold standard dataset (Figure S6e-f). After the outliers were identified, a visual examination of the sets of radiographs with the highest discordance between submitted scores and SvH scores was performed to adjudicate whether the algorithm-generated score or the expert-curated score was more accurate. The adjudication was performed by a board-certified musculoskeletal radiologist (MBF), who reviewed the 663 (201 JSN, 462 erosion) discrepant measurements to determine whether the original measurements were indeed inaccurate, and if they were, to revise these joint scores for JSN and erosion to more accurately reflect 'truth'. We created an in-house Matlab visualization package to overlay the scores on the images and increase the speed of the evaluation manual revision process. Using this approach, we were able to visualize and evaluate the accuracy of the gold-standard dataset. The corrected gold-standard was used for all post-challenge analysis.

Finalist team methods (write ups)

All finalist teams applied deep-learning-based methods in both segmentation (when applicable) and joint score prediction steps. The teams manually located and marked the joint regions to train the segmentation model in the segmentation step. The deep-learning-based segmentation model may directly learn to draw the joint bounding boxes or the pre-defined points marking the joint. Pre-trained deep-learning-based models, including DenseNet,[1] U-Net,[2] ResNet,[3] Efficient-Net,[4] YOLO v3,[5] and Faster RCNN,[6] were intensively retrained and repurposed for the DREAM Challenge dataset. Besides, the following practice improved the model performance

- *XGBoost*[7] is a regularization gradient boosting software library that implements deep learning algorithms' core optimizer. Compared to other gradient boosting implementations, XGBoost is explicitly for distributed computing, which means multiple computers can solve the optimization together. This implementation has the advantage in cloud computing and supercomputing platforms, such as in Cheaha.
- *Hyperparameter selection*. The performance of the deep-learning-based model depends on the hyperparameters (such as initial weights), which have to be set before computing the models. This step

© 2022 Sun D et al. *JAMA Network Open.*

requires manual decisions from each team. In practice, each team prepared some hyperparameter sets. They randomly split the training data into two parts. One part was used to compute the model with the selected hyperparameter set; and the other was used to test and compare the performance of the hyperparameter sets.

- Ensemble model. Some teams trained multiple independent joint score prediction models, then combined these models' results to determine the final joint score. There were two approaches to combine the models' results. The averaging/voting approach considered all models equal to each other. Then, the final score was determined by averaging all results. The sequential approach has one classification model and some regression models. When combining, the classification model determines whether the joints have damage; here, '0' means that the joints have no damage, and the final joint scores are 0. Otherwise, the regression models, which predict the damaged joint scores from 1 to 5, determine the final joint score.
- *VGG*[8] is a software library to create and train a new deep-learning model. The VGG-based deep learning network has 16 or 19 layers. As mentioned above, this library is used when the team decides not to retrain and repurpose the pre-trained models.
- *Autoencoder* is a deep-learning architecture that matches two homogeneous data sets, such as translation between two languages or synthesizes the data. The autoencoder synthesizes 'fake' data such that the classification algorithms may not differentiate between the 'fake' and the 'original data. The deep autoencoder includes an input 'original' data layer, an output 'fake' data layer, and many layers between. The center layer, which is also called the encoded layer, has the smallest size. The result at this layer is the most compacted the highest-quality representation of the original data yet; therefore, the encoded layer is used as features for later machine learning models.

Shirin Team

We first trained models to rotate/flip all images into the same 90 degree orientation, allowing for differently oriented input images and also effectively doubling the training set size (left and right images).

Joint centers were then manually marked on the training set, and this was used to train models to locate hand and foot joints on radiographs. For the finger and toe joints each joint was marked separately. The 12 wrist joints were grouped into 4 center points representing 3 joints each.

Joint sub-images were cut out using the above joint location models by taking a square image at the predicted joint center that was a fixed percentage of the original images (12.5% for fingers and toes, 15% for thumb/big toe, 25% for wrist). These joint sub-images were used to train models to predict erosion and joint narrowing scores. Groups of joints were merged to increase training data per model; for example, a single model was trained to predict all finger joint erosion scores, resulting in several thousand training examples for that model.

In addition to more commonly used data augmentation transforms (brightness, zoom, rotation, etc), perspective warps were used to simulate joints held at different angles to the X-ray plane. Gaussian blurring[9] was also found to improve performance, particularly on erosion prediction. Joints with more severe damage were duplicated to help with imbalance in the training set.

Models for orientation and joint center location used a pretrained Resnet34 architecture with 256x256 image size. Models for joint damage prediction used a pretrained Densenet201 architecture,[1] also with image size 256x256 (note this was the size of the joint sub-images cut from the original images as described).

Five-fold cross-validation was used to manually select the best hyperparameters for joint damage prediction models. Single models were then trained using the best hyperparameter settings and all training data and used for the final evaluation set prediction. The final SvH score was added from all joint scores.

HYL-YFG (Hongyang Li and Yuanfang Guan) Team

All images were uniformly scaled to 1024x1024-pixel size. Each joint in the image was manually marked by a 30x30 pixel bounding box. Image background noise filtering was applied. Two types of deep-learning models were trained. First, for image segmentation, there was a model trained for each joint. Each model was retrained using the U-Net (kernel size 7) pre-trained model and the joint bounding-box sub-images. Each U-Net[2] used three input channels: the original, gray-scaled image, the post-quantile-normalization image, and the post-noise-filtering image. Second, for joint score prediction, new deep-learning-based models were trained using seven input channels. The input channels include: (1) the overall original black-and-white image, (2) the overall image after quantile

normalization, (3) the overall image after rescaling to the range between 0 and 255, (4) the overall image after filtering out background and quantile normalization, (5) the overall image after Gaussian normalization, (6) the bounding-box joint image after rescaling to the range between 0 and 255 only based on image patches, and (7) the bounding-box joint image after Gaussian normalization. Four joint score prediction models were built using deep learning to predict: (1) joint space narrowing in hand, (2) joint space narrowing in foot, (3) joint erosion in hand, and (4) joint erosion in foot. To predict the final patient SvH score, the joint scores were used as features to train an ensemble random forest model. The random forest consisted of 500 random decision trees.

In the final evaluation round, all the above input channels were computed. The U-Net retrained segmentation model automatically located the 30x30 pixel bounding box for each joint. Each joint score was predicted using the bounding boxes and image channels above as features. These predicted joint scores were fed toward the random forest model to predict the patients' SvH scores.

Csabaibio Team

From the challenge training image, horizontal flipping, 10-degree random rotating, and randomly changing 5% of the brightness and contrast were applied to increase the training set size and improve the model robustness. In each image, the joints were manually marked by rectangle bounding boxes. The deep-learning-based and pre-trained Mask-RCNN[10] and ResNet-50[3] were combined and trained using the bounding-box sub-images for image segmentation. All sub-images were rescaled to the size of 256x266 pixels. For joint score prediction, each joint score was the average of 12 independent deep-learning-based models. These 12 models were retrained from the following pre-trained models (citation): ResNet-34 (6 models), ResNet-50 (4 models), and Vgg16 (2 models). The training hyperparameters were manually selected by 5-fold cross-validation.

In the final evaluation round, the segmentation model automatically located the bounding boxes for the joints in each image. The bounding-box sub-images were rescaled to the size of 256x266 pixels. Then, the ensemble prediction model used the bounding-box images to make the joint score prediction. The patient's final SvH score was added from all joint scores.

© 2022 Sun D et al. *JAMA Network Open.*

Nc717 Team

The joints in the training images were manually marked by rectangle bounding boxes and labeled. The bounding boxes were used to train two segmentation models: one for hand images and the other for foot images. In segmentation, the RetinaNet/ResNet[3,11] pre-trained model was retrained using the joint images. In each joint score prediction, five models were retrained from the pre-trained Efficient-Net model.[4] For each joint score, the final score was averaged from the results of these 5 models.

In the final evaluation round the segmentation model automatically located the bounding boxes for the joints in each image. Then, the ensemble prediction model used the bounding-box images to make the joint score prediction. The patient's final SvH score was added from all joint scores.

Zs_aicoe Team

The joints in the training images were manually marked by rectangle bounding boxes and labeled using YOLO v3 software.[5] The images and bounding boxes were used to retrain YOLO v3 model for segmentation.[5] Segmentation retraining continued until the segmentation 'Intersection over union' metric reached 0.9 for hand images and 0.88 for foot images. Joint sub-images inside the bounding boxes were randomly rotated and shifted by a small amount; this created more images to train the joint score model later. Different pre-trained models and ensemble approaches were applied to train models that predict different types of joint scores.

- For erosion joint scores, the pre-trained ResNet-50 model was retrained into three models. The ResNet-50[3] classification-based model considered each joint score is a binary class: 0 (no damage) versus other (with damage, erosion score from 1 to 5). The ResNet-50 regression-based model considered each joint score as a continuous number. So does the ResNet-XGBoost[7] regression-based model technique (citation). To combine three model results into one erosion joint score, first, the classification-based result was examined. If the classification-based model predicted class 0, then the final erosion joint score is 0. Otherwise, the average result of the two regression-based models was used to determine the discrete damage score.
- For narrowing scores, only the ResNet-XGBoost[7] regression-based model was retrained and predicted the joint score. No ensemble approach was applied. In the final evaluation round, the segmentation models automatically located the bounding boxes for the joints in each image. The ensembled ResNet-50 and

© 2022 Sun D et al. *JAMA Network Open.*

ResNet-XGBoost models predicted the erosion scores; the ResNet-XGBoost model predicted the narrowing scores. The patient's final SvH score was added from all joint scores.

NAD Team

From the challenge training image, horizontal flipping, small-degree random rotating, small shifting was applied to increase the training set size and improve the model robustness. Thirty-four bounding boxes were manually marked in each patient's image, including 12 feet-joint boxes (6 left, 6 right), 20 finger-joint boxes (10 left, 10 right), and 2 boxes that covered the patient's wrist joints (1 left, 1 right). The images and bounding boxes were used to retrain the Faster RCNN pre-trained model for segmentation.[6] In predicting the joint scores, 15 independent models were retrained from the EfficientNet pre-trained model.[4] These 15 models were created by 15 rounds of random splitting in the training set. For each joint score, the final score was determined by voting the output of these 15 models.
In the final evaluation round, the segmentation models automatically located the bounding boxes for the joints in each image. The ensembled 15 models predicted the joint scores using the sub-images inside the bounding boxes. The patient's final SvH score was added from all joint scores.

Gold Therapy Team

From the challenge training image, horizontal flipping, small-degree random rotating, and randomly changing a small amount of the brightness and contrast were applied to increase the training set size and improve the model robustness. Each image was marked by a predetermined set of anchor points, as showed in https://www.synapse.org/#!Synapse:syn21499370/wiki/604451. These anchor points located the joints' locations in the image and were the target of the pre-trained ResNet segmentation model. Using the predetermined anchor points as the reference, after the ResNet-based3 segmentation model found these corresponding points in the patient images, the model could uniformly draw the bounding boxes, scale, and rotate the bounding-box sub-images to the same size and orientation. For each joint damage score, the regression-based pre-trained ResNet-35 and RestNet-50 models[3] were retrained and enhanced by XGBoost.[7] Models' hyperparameters were determined empirically using hyperopt.[12]

In the final evaluation round, the segmentation models automatically located the anchor points in each image. The joint bounding boxes were automatically extracted, scaled, and rotated from the detected anchor points. The

ensembled ResNet-35 and RestNet-50[3] models predicted the joint scores by averaging the output of these models. The patient's final SvH score was added from all joint scores.

Alpine Lads Team

Each image was marked by a predetermined set of landmark points, as showed in https://www.synapse.org/#!Synapse:syn21610007/wiki/604496. These landmarks located the joints' locations in the image and were the target of the VGG-based model.[8] The landmarks define bounding-box regions that surround the joints in the images. These bounding-box sub-images were rescaled to the size of 224x224 pixels and fed to the extended-VGG model for each joint score prediction. Before training, extended-VGG model,[8] the sub-images were randomly zoomed by a small amount to increase the number of training samples. In predicting the joint damage score, the extended-VGG model consisted of two parts. The first part was VGG-based binary classification, which determined whether the joint had damage (0 for no damage, 1 for joint score from 1 to 5). Images that received the classification of '0' had the joint score of 0. Meanwhile, images that received the classification of '1' were further fed to the VGG-based regression model, where the model result determined the joint damage score.

In the final evaluation round, the segmentation models automatically located the landmarks in each image. The 224x224-pixel joint bounding boxes were automatically extracted from the detected landmarks. The two-stage VGG-extended model predicted the joint scores by averaging the output of these models. The patient's final SvH score was added from all joint scores.

RYM Team

All training images were cropped to remove unnecessary background. For the hands, the bottom 1/7 of each image was removed. This mostly removed the beginnings of the ulna and radius bones and part of the wrist. For the feet, the bottom ¼ of each image was removed. This did not remove any of the joints involved in scoring as the toes are found nearer the top of the feet. After cropping, the training images were scaled to the size of 1500 x 1200 pixels. A U-Net[2] model was trained to remove the nonuniform background intensity, which was considered noise prior to segmentation. Then, the joints in the image were manually marked by rectangle bounding boxes. Two pre-trained YOLOv3[5] segmentation models were retrained for segmenting these joint bounding boxes; one segmented the hand

© 2022 Sun D et al. *JAMA Network Open.*

images, and the other segmented the foot images. To predict each joint score, a VGG-based model was created and trained using the corresponding joint sub-images.

In the final evaluation round, the U-Net model above filtered out the image noise. Then, the YOLOv3 segmentation models automatically located the joint bounding boxes in each image. The VGG-based models predicted the joint scores. The patient's final SvH score was added from all joint scores.

CU_DSI_RA2_Challenge Team

The joints in the training images were manually marked by rectangle bounding boxes and labeled. For image segmentation, the deep-learning-based and pre-trained FasterCNN[6] was retrained using the bounding-box sub-images. The bounding boxes were scaled to the size of 224x224 pixels. For predicting each joint score, the pre-trained EfficientNet[4] was retrained using the 224x224-pixel sub-images. Before training, the sub-image data were augmented by randomly cropping, horizontal flipping, rotating, and distorting by a small amount.

In the final evaluation round, the FasterCNN segmentation model automatically located the 224x224-pixel joint bounding boxes in each image. The EfficientNet models predicted the joint scores. The patient's final SvH score was added from all joint scores.

KichuDL Team

Contrast Limited AHE[13] was applied in all images to remove the background noise. Then, all images were scaled to the size of 128x128 pixels. Then, a deep autoencoder was trained from these 128x128-pixel images. In this autoencoder, the input had the size 128x128, which corresponded to the scaled input image. The output layer had the same size as the input layer, which corresponded to the synthetic image. The middle layer, which was the smallest one, had the size of 32x32. After passing through the autoencoder, the results at the 32x32 middle layer were used to train other deep-learning models to predict the joint scores. Here, for each joint score, 20 independent models were trained and ensembled.

© 2022 Sun D et al. *JAMA Network Open.*

In the final evaluation round, first, Contrast Limited AHE filtered the image background noise. Second, the image was fed into the autoencoder from the input to the middle layer. Third, the output at the middle layer was fed into the deep learning models to predict the joint scores. The patient's final SvH score was added from all joint scores.

Aboensis V Team

All images were scaled to the size of 800x800 pixels. The joints in the training images were manually marked by rectangle bounding boxes and labeled using YOLO v3 software.[5] The images and bounding boxes were used to retrain YOLO v3 model for segmentation. Then, to predict each joint score, a pre-trained YOLO v3 model was retrained using the bounding-box sub-images.

In the final evaluation round, the YOLO v3 segmentation model automatically located the joint bounding boxes in each image. The YOLO v3 prediction models predicted the joint scores. The patient's final SvH score was added from all joint scores.

Zbigniew Wojna Team

The joints in the training images were manually marked by rectangle bounding boxes and labeled. The images and bounding boxes were used to retrain UNet[2] model for segmentation. Then, to predict each joint score, 8 pre-trained UNet models were retrained using the bounding-box sub-images. The final joint score was the average of these 8 models.

In the final evaluation round, the UNet segmentation model automatically located the joint bounding boxes in each image. Eight UNet prediction models predicted and ensembled the joint scores. The patient's final SvH score was added from all joint scores.

eReferences

1 Iandola F, Moskewicz M, Karayev S, Girshick R, Darrell T, Keutzer K. DenseNet: Implementing Efficient ConvNet Descriptor Pyramids. *ArXiv14041869 Cs* 2014; published online April 7. http://arxiv.org/abs/1404.1869 (accessed Jan 26, 2022).

© 2022 Sun D et al. *JAMA Network Open.*

2 Ronneberger O, Fischer P, Brox T. U-Net: Convolutional Networks for Biomedical Image Segmentation. *ArXiv150504597 Cs* 2015; published online May 18. http://arxiv.org/abs/1505.04597 (accessed Aug 26, 2021).

3 Targ S, Almeida D, Lyman K. Resnet in Resnet: Generalizing Residual Architectures. *ArXiv160308029 Cs Stat* 2016; published online March 25. http://arxiv.org/abs/1603.08029 (accessed Jan 26, 2022).

4 Tan M, Le Q. EfficientNet: Rethinking Model Scaling for Convolutional Neural Networks. In: Proceedings of the 36th International Conference on Machine Learning. PMLR, 2019: 6105–14.

5 Tian Y, Yang G, Wang Z, Wang H, Li E, Liang Z. Apple detection during different growth stages in orchards using the improved YOLO-V3 model. *Comput Electron Agric* 2019; **157**: 417–26.

6 Ren S, He K, Girshick R, Sun J. Faster R-CNN: Towards Real-Time Object Detection with Region Proposal Networks. *ArXiv150601497 Cs* 2016; published online Jan 6. http://arxiv.org/abs/1506.01497 (accessed Jan 26, 2022).

7 Chen T, Guestrin C. XGBoost: A Scalable Tree Boosting System. In: Proceedings of the 22nd ACM SIGKDD International Conference on Knowledge Discovery and Data Mining. San Francisco, CA, USA: Association for Computing Machinery, 2016: 785–94.

8 Sengupta A, Ye Y, Wang R, Liu C, Roy K. Going Deeper in Spiking Neural Networks: VGG and Residual Architectures. *Front Neurosci* 2019; **13**. DOI:10.3389/fnins.2019.00095.

9 Carreira-Perpiñán MÁ. Fast nonparametric clustering with Gaussian blurring mean-shift. In: Proceedings of the 23rd international conference on Machine learning. New York, NY, USA: Association for Computing Machinery, 2006: 153–60.

10 Yu Y, Zhang K, Yang L, Zhang D. Fruit detection for strawberry harvesting robot in non-structural environment based on Mask-RCNN. *Comput Electron Agric* 2019; **163**: 104846.

11 Wang Y, Wang C, Zhang H, Dong Y, Wei S. Automatic Ship Detection Based on RetinaNet Using Multi-Resolution Gaofen-3 Imagery. *Remote Sens* 2019; **11**: 531.

© 2022 Sun D et al. *JAMA Network Open.*

12 Hyperopt Documentation. http://hyperopt.github.io/hyperopt/.

13 Yadav G, Maheshwari S, Agarwal A. Contrast limited adaptive histogram equalization based enhancement for real time video system. In: 2014 International Conference on Advances in Computing, Communications and Informatics (ICACCI). 2014: 2392–7.

© 2022 Sun D et al. *JAMA Network Open.*

eFigure 1. RA2-DREAM Challenge Timeline

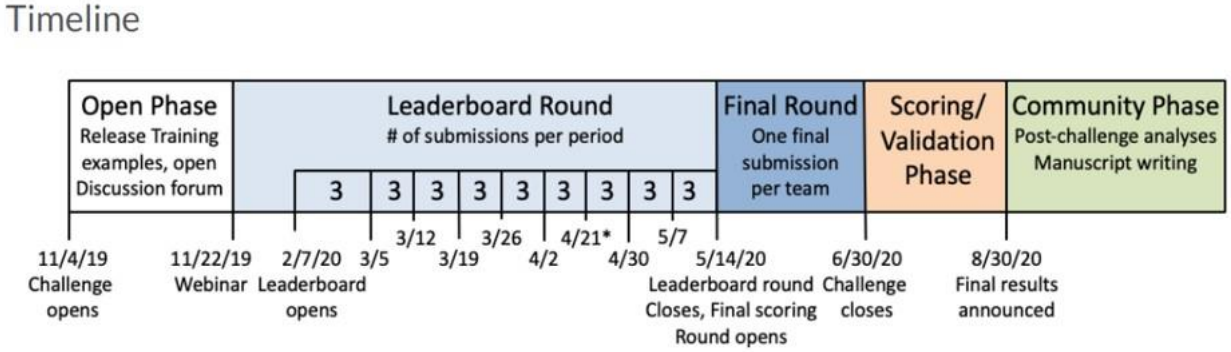

eFigure 2. Distribution of Individual and Ensembled Predictions

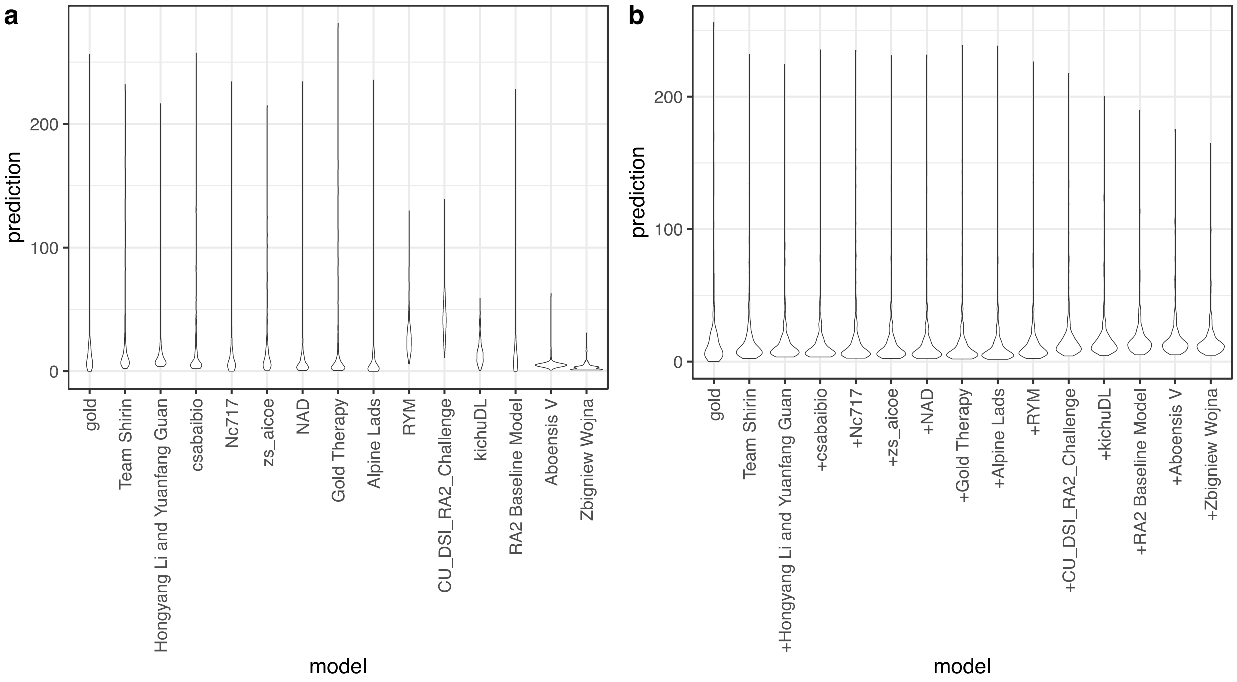

a. The distribution of the submitted predictions (SC1,2,3 measurements) for each team. Compared to better-ranked predictions, the RYM model predicted greater damage for all patients. b. The distribution of the ensembled predictions (SC1,2,3 measurements). When ensembled, the addition of the RYM model made the distribution of the predictions closer to that of the gold standard.

© 2022 Sun D et al. *JAMA Network Open.*

eFigure 3. Spearman Correlation Between Top Final Round Predictions

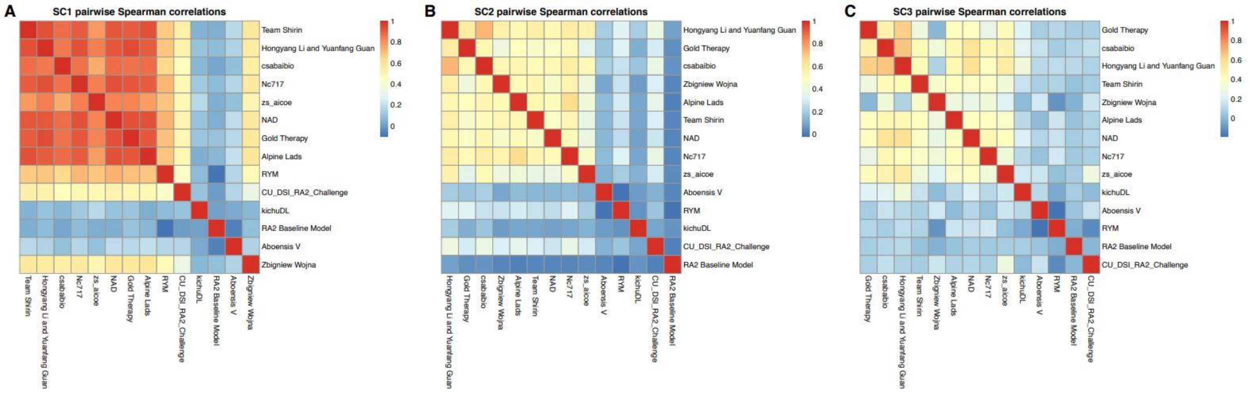

The pairwise Spearman correlation was determined for all final round predictions in SC1 (a), SC2 (b), and SC3 (c). The predictions are sorted by highest to lowest performance in each subchallenge from left to right and from top to bottom.

eFigure 4. Performance of Ensembled Models

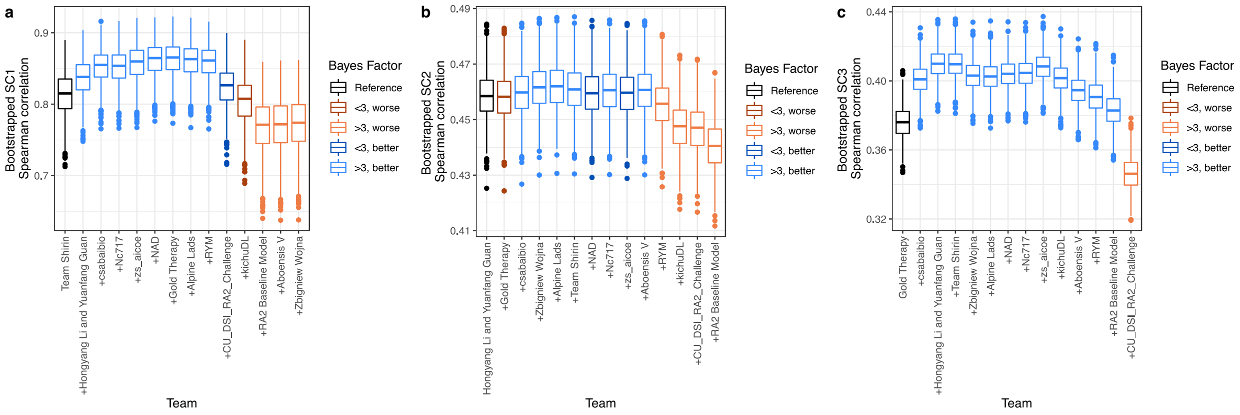

The analysis described for Figure 3 was repeated using Spearman correlation as the performance metric instead of weighted RMSE. Using this metric, we observed improved performance (Bayes < 3, light blue) for many of the ensembled models in a. SC1, b. SC2, and c. SC3.

© 2022 Sun D et al. *JAMA Network Open.*

eFigure 5. Summary of Approaches and Methods Used By Participated Teams in 3 Subchallenges

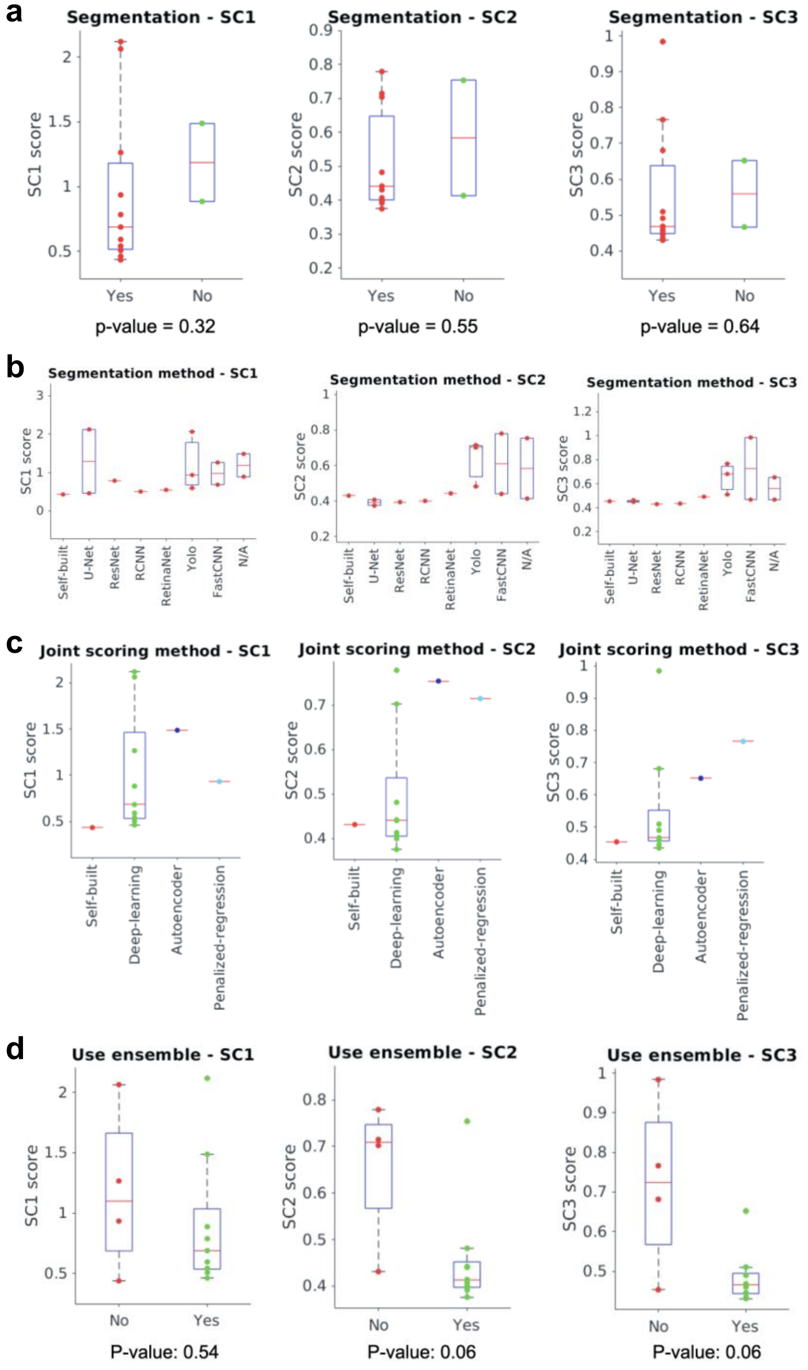

a. Impact of segmentation on the subchallenge scores. Teams using vs. not using segmentation methods were compared. b. Impact of segmentation algorithms on the subchallenge scores. Participants self-built segmentation method, U-net, ResNet, RCNN, RetinaNet, Yolo, FastCNN and without any segmentation method were compared. c. Impact of scoring algorithm on the subchallenge scores. Participants self-built algorithm, Deep-learning, Autoencoder and Penalized-regression were compared. d. Impact of using ensemble models on the subchallenge scores. Teams using ensemble models (n=8) vs not using ensemble models (n=5) were compared. We used the Kruskal-Wallis (KW) test for all the comparison. The p value < 0.05 is considered significant.

© 2022 Sun D et al. *JAMA Network Open.*

eFigure 6. Identification and Correction of Criterion Standard Outliers

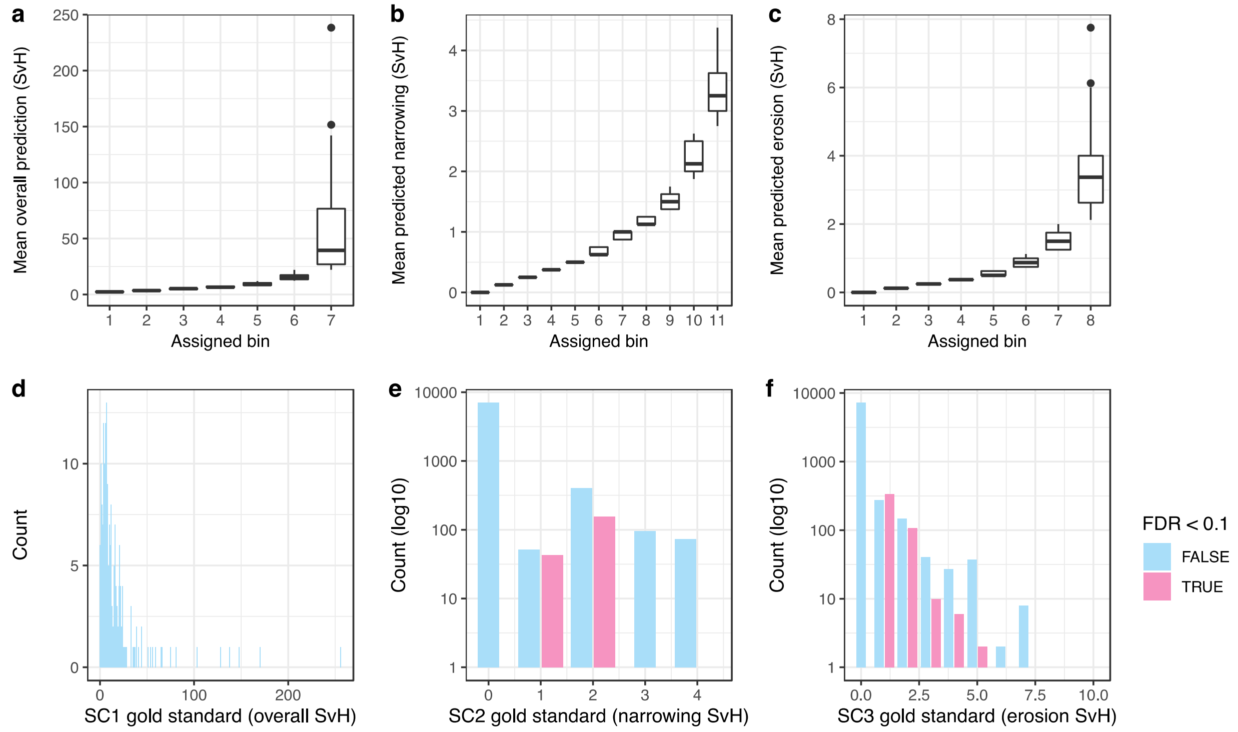

a-c. Averaged predictions from top methods were used to assign each measurement to a bin and identify potential outliers (see supplemental methods) in the gold standard dataset for SC1, 2 and 3. d-f. False discovery rate (FDR)-adjusted empirical p-values were calculated for each gold standard measurement by comparing them to the rest of the bin they were assigned to. We did not identify any potential outliers in SC1 (overall SvH) but identified several in SC2/SC3 measurements. The potential outlier measurements and the images were reviewed by an expert and, if necessary, corrected.

© 2022 Sun D et al. *JAMA Network Open.*
