# Supplementary material for: A Crowdsourcing Approach to Develop Machine Learning Models to Quantify Radiographic Joint Damage in Rheumatoid Arthritis
Source: JAMA Netw Open. 2022 Aug 29;5(8):e2227423. doi: 10.1001/jamanetworkopen.2022.27423 (PMC9425151; doi:10.1001/jamanetworkopen.2022.27423)
Supplement: Supplement 2. — The RA2-DREAM Challenge Community Members [file jamanetwopen-e2227423-s002.pdf]

Supplemental Online Content: Nonauthor Collaborators
*First name, last name, and suffix (if applicable) are required and will appear in PubMed.

***Group Name(s): The RA2-DREAM Challenge Community**							
***First Name and Middle Initial(s)**	***Last Name**	***Suffix (eg, Jr, III)**	Academic Degrees	Institution	Location (city, state/province, country)	Role or Contribution, eg, chair, principal investigator	Group (if more than 1 Group listed in the byline) and/or Subgroup (eg, Steering Committee)
Zbigniew	Wojna			Tensorflight, Inc	350 5th Avenue, Suite 4215, New York, NY 10118, USA	non-author collaborator	NA
Anna	Krason			University College London	University College London, Gower St, London WC1E 6BT	non-author collaborator	NA
YanMing	Tan			NUS Department of Statistics and Data S	21 Lower Kent Ridge Rd, National University of Singapore, Singapo	non-author collaborator	NA
RaphaelHaoChong	Quek			NUS Department of Statistics and Data S	21 Lower Kent Ridge Rd, National University of Singapore, Singapo	non-author collaborator	NA
Neelambuj	Chaturvedi			ZS Associates	Safina Towers, Bengaluru, India	non-author collaborator	NA
Michael	Stadler			The University of Manchester	Medicine and Health, The University of Manchester, UK	non-author collaborator	NA
Chenfu	Shi			The University of Manchester	Medicine and Health, The University of Manchester, UK	non-author collaborator	NA
Krishnakumar	Vaithinathan			Karaikal Polytechnic College	Varichikudy, Karaikal-609609, Puducherry, India	non-author collaborator	NA
Julian	Benadit			School of Engineering and Technology, (	Kengeri campus, Kanmanike, Bangalore-560074, India	non-author collaborator	NA
Duc	Tran			University of Nevada	University of Nevada, Reno, NV 89557, USA	non-author collaborator	NA
Tin	Nguyen			University of Nevada	University of Nevada, Reno, NV 89557, USA	non-author collaborator	NA
Alexander	Biehl			University of Turku and Åbo Akademi U	University of Turku and Åbo Akademi University, Turku, Finland	non-author collaborator	NA
Mehrad	Mahmoudian			University of Turku and Åbo Akademi U	University of Turku and Åbo Akademi University, Turku, Finland	non-author collaborator	NA
Sami	Pietilä			University of Turku and Åbo Akademi U	University of Turku and Åbo Akademi University, Turku, Finland	non-author collaborator	NA
Tomi	Suomi			University of Turku and Åbo Akademi U	University of Turku and Åbo Akademi University, Turku, Finland	non-author collaborator	NA
Mikko S	Venäläinen			University of Turku and Åbo Akademi U	University of Turku and Åbo Akademi University, Turku, Finland	non-author collaborator	NA
Laura L	Elo			University of Turku and Åbo Akademi U	University of Turku and Åbo Akademi University, Turku, Finland	non-author collaborator	NA
Chenguang	Xue			ZS Associates Inc	1560 Sherman Ave, Evanston, IL, 60201	non-author collaborator	NA

Supplemental Online Content: Nonauthor Collaborators
*First name, last name, and suffix (if applicable) are required and will appear in PubMed.

***First Name and Middle Initial(s)**	***Last Name**	***Suffix (eg, Jr, III)**	Academic Degrees	Institution	Location (city, state/province, country)	Role or Contribution, eg, chair, principal investigator	Group (if more than 1 Group listed in the byline) and/or Subgroup (eg, Steering Committee)
Akshat	Shreemali			ZS Associates Inc	1560 Sherman Ave, Evanston, IL, 60201	non-author collaborator	NA
Srinivas	Chilukuri			ZS Associates Inc	1560 Sherman Ave, Evanston, IL, 60201	non-author collaborator	NA
Khanh-Tung	Nguyen-Ba			Columbia University	Room 1005 SSW, MC 4690, 1255 Amsterdam Avenue, New York, [illegible]	non-author collaborator	NA
Jay Ji-Hyung	Ryu			Columbia University	Room 1005 SSW, MC 4690, 1255 Amsterdam Avenue, New York, [illegible]	non-author collaborator	NA
Rui	Bai			Columbia University	Northwest Corner, 550 W 120th St, #1401, New York, NY 10027	non-author collaborator	NA
Yilin	Wu			Columbia University	Room 1005 SSW, MC 4690, 1255 Amsterdam Avenue, New York, [illegible]	non-author collaborator	NA
Yingnan	Wu			Columbia University	Northwest Corner, 550 W 120th St, #1401, New York, NY 10027	non-author collaborator	NA
Xiaofu	He			Columbia University	Northwest Corner, 550 W 120th St, #1401, New York, NY 10027	non-author collaborator	NA
